# The Use of Neural Networks in Identifying Error Sources in Satellite-Derived Tropical SST Estimates

**DOI:** 10.3390/s110807530

**Published:** 2011-07-29

**Authors:** Yung-Hsiang Lee, Chung-Ru Ho, Feng-Chun Su, Nan-Jung Kuo, Yu-Hsin Cheng

**Affiliations:** 1 Department of Marine Environmental Informatics, National Taiwan Ocean University, Keelung 20224, Taiwan; E-Mails: D93840002@ntou.edu.tw (Y.-H.L.); c0021@ntou.edu.tw (N.-J.K.); B96810053@ntou.edu.tw (Y.-H.C.); 2 Department of Marine Biotechnology and Resources, National Sun Yat-sen University, Kaohsiung 80424, Taiwan; E-Mail: fengchunsu@gmail.com

**Keywords:** infrared sensor, data mining, neural network, sea surface temperature, tropical pacific

## Abstract

An neural network model of data mining is used to identify error sources in satellite-derived tropical sea surface temperature (SST) estimates from thermal infrared sensors onboard the Geostationary Operational Environmental Satellite (GOES). By using the Back Propagation Network (BPN) algorithm, it is found that air temperature, relative humidity, and wind speed variation are the major factors causing the errors of GOES SST products in the tropical Pacific. The accuracy of SST estimates is also improved by the model. The root mean square error (RMSE) for the daily SST estimate is reduced from 0.58 K to 0.38 K and mean absolute percentage error (MAPE) is 1.03%. For the hourly mean SST estimate, its RMSE is also reduced from 0.66 K to 0.44 K and the MAPE is 1.3%.

## 1. Introduction

Sea Surface Temperature (SST) is an important factor in ocean processes with a major impact on weather and climate [1]. Since the 1980s, infrared and microwave sensors onboard satellites have been widely used to observe the spatial and temporal distributions and variations of SST in the World’s oceans. Satellite measured SST provides a synoptic and high frequency views of the ocean that is not easily achieved by ships or buoys. Multi-channel Sea Surface Temperature (MCSST) is the first algorithm to derive SST from satellite infrared sensors. It is derived from radiances collected by the Advanced Very High Resolution Radiometer (AVHRR) sensor carried onboard the National Oceanic and Atmospheric Administration (NOAA) polar orbiting satellites. The MCSST algorithm is a kind of linear regression method. There are three approach methods for the regression method including split window, dual window, and triple window [2]. A revised MCSST algorithm is introduced to include the effect of variable satellite zenith angle [3]. The overall error of SST using the MCSST algorithm is around 0.6–0.7 K [4–6]. To reduce the error, a further improvement of the MCSST algorithm, the nonlinear SST (NLSST), is developed. The NLSST accounts for a minor non-linearity in water vapor by including a first-guess SST that is a surrogate for total water vapor amounts [7–10]. By using these algorithms, NOAA has been continuously providing research quality SST data since 1981. However the infrared sensors onboard the satellites cannot look through clouds. After removing cloud covered regions of satellite sampling, the available data are less than 15% [1]. On the other hand, geostationary satellite, different from polar orbiting satellite always flies above the same location at the equator. It excels with its higher temporal resolution than that of the polar orbiting satellite. This enables us to obtain a better cloud free SST dataset with integration of time but the accuracy of SST is less than that derived from polar orbit satellites. The algorithm for retrieving SST from geostationary satellite is the same as the algorithm used in polar orbiting satellites. However, the higher system noise levels of SST derived from geostationary satellites are larger than those derived from polar orbiting satellites [11]. Previous studies [12–14] have pointed out that the insolation may be the main factor to affect the accuracy of SST derived from geostationary satellites because of different observation of insolation areas at the same time. Therefore, the main purposes of this study are to figure out major factors of causing the error of Geostationary Operational Environmental Satellite (GOES) SST estimates in the tropical Pacific and to improve the accuracy of the GOES SST products.

To achieve these purposes, we use an artificial neural network (ANN) algorithm of data mining technique in this study. Data mining is a process of automatically or semi-automatically analyzing data from different perspectives to discover useful information [15]. It is also the process of finding correlations or patterns in a group of data [16–18]. Recent literatures reveal that there are various soft computing techniques employed in several real-life applications in different fields. Cheng *et al.* [19] combined a fuzzy optimal model with a genetic algorithm to solve multi-objective rain-full-runoff model calibration. Their research not only can improve forecast accuracy but also is an efficient and robust means. Muttil and Chau [20] derived a good prediction of long-term trends in algal biomass by using ANN and genetic program. Xie *et al.* [21] used a hybrid adaptive time-delay ANN model on single factor time series, such as sunspot. The results showed their model is capable of capturing potential information and relationship in the analysis of time serial data. Liu *et al.* [22] predicted the long-term discharges in the Man Wan Hydropower Scheme with a support vector machine. They demonstrated that the support vector machine is a very useful tool for problem prediction. Zhang and Chau [23] developed a novel multi-sub-swarm optimization to find multi-solutions, and the method can improve the generalization performance for multilayer ensemble pruning model. Furthermore, Chau [24] demonstrated that the particle swarm optimization technique is a good alternative algorithm in the ANN training procedure. ANN has been widely used in developing satellite retrieval procedures [25,26]. The concept of artificial neurons was first introduced by McCulloch and Pitts [27], and applications of Back-Propagation Network (BPN) algorithm for feed-forward ANNs appeared in 1986 [28]. In previous studies, BPN is the most widely used model in ANN, and it is one of the most frequently cited data mining method algorithms [29].

## 2. Data

### 2.1. In Situ Data

**Figure 1.**
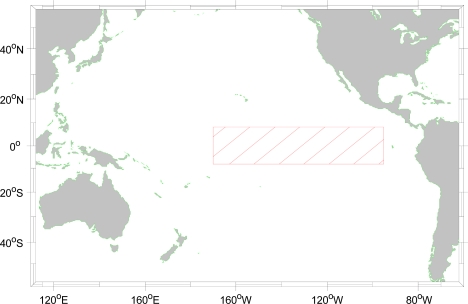
The red rectangle represents the study area from 8°S to 8°N in latitude and from 95°W to 170°W in longitude.

To evaluate the accuracy of data products derived from satellite infrared sensors, *in situ* measurement is necessary. The buoy data from the Tropical Atmosphere Ocean (TAO) Project in the tropical Pacific Ocean (http://www.pmel.noaa.gov/tao/data_deliv) have been used for comparison and validation the satellite data. For example, Murray *et al.* [12] used TAO SST and wind data to validate the ATSR (Along Track Scanning Radiometer) satellite data. Neeaj *et al.* [30] used the TAO data to validate the quality of QuikSCAT and NCEP (National Centers for Environmental Prediction) data. Wu [31] and Legeckis and Zhu [32] applied TAO 1 m depth of the accuracy of SST can be 0.01 K. Chambers *et al.* [33] measured heat content of the Pacific equatorial region, using TOPEX altimeter and TAO data. Therefore, in this study we also used TAO data as the ground truth to compare and validate our results. The study area is from 8°N to 8°S in latitude and from 95°W to 170°W in longitude, as shown in Figure 1. The time span of the data is from May 2003 to December 2007. The TAO data at some stations have high sampling rate of 10 min. We have averaged the 10-min data into hourly and daily datasets including wind speed and relative humidity. We also compute the standard deviation of the data products when we perform the average.

The TAO wind direction dataset is a 0 to 360 value data. The tropical Pacific the easterly direction predominates. Following the concept of data mining of generalization, we make wind direction data abstract and aggregated, which simplified wind directions to four directions by 45-degree angels on both sides to make model efficient. To meet the limitations of the ANN, the wind direction is assigned into four sets as north wind (315°–44°), –east wind (45°–134°), south wind (135°–224°) and west wind (225°–314°).

### 2.2. Satellite Data

The GOES SST used in this study is a level 3 product and archives from the Physical Oceanography Distributed Active Archive Center (PO.DAAC). The spatial resolution of this product is 6 km and temporal resolution is one hour. In order to make a comparison with *in situ* measurement, the satellite data have been averaged with 5 × 5 pixels of the center at the location of *in situ* measurement.

To understand the impact of satellite zenith angle to the GOES SST product, we include it as a parameter for analysis. The azimuth angle between the in-situ data point and GOES satellite is calculated by the spherical trigonometric equation as:

(1) $$\text{cos}\theta =-\frac{a_{e}-(a_{e}+H)\text{cos}\psi }{(a_{e}+H)\text{sin}\psi }$$

where *a_e_* is the radius of the Earth, *H* the height of the GOES satellite remains from the Earth, *ψ* is the Earth’s center to the GOES satellite and the buoy included angle, and *θ* is the azimuth angle. Since BPN only deals with numerical data, the wind direction information is set to numbers as aforementioned based on mean tests of wind direction angles.

### 2.3. Data Process

Original match-up data are hourly mean data. As indicated by Zeng *et al.* [34], the Sun would heat up the sea surface to form a hotter skin during daytime, while the difference between diurnal and nocturnal SST could be as great as 3 K, normally 1 K [35]. Hourly mean data in this study are divided as diurnal (08:00–17:00 local time) and nocturnal (19:00–05:00 local time). To reduce the solar effect, the nocturnal data are used for model analysis. In most cases, the scope of the different variables varies significantly in neural network input layer variables. Series of small variable input variables influence on the network will be lower than the input variables of a larger series. All variables in this study are explained in Table 1. In the in-situ data, net longwave radiation flux ( $X_{i}^{\mathrm{nlwrs}}$), offering daily average datasets which seven variables have been used of eight variables, substitutes hourly datasets. These have been normalized between −1 and 1, by using Equations (2) and (3) as

(2) $$P_{\mathrm{new}}=\frac{2(P-P_{\text{min}})}{P_{\text{max}}-P_{\text{min}}}-1$$

(3) $$P=0.5(P_{\mathrm{new}}+1)(P_{\text{max}}+P_{\text{min}})+P_{\text{min}}$$

The ANN formulation requires three sets of data for training, testing, and verification (Table 2). Total of 70% of daily mean data are randomly picked and sets as training data, among which, 50% is used for training, 25% for model testing and correction; and 25% for model verification. The remaining 30% (5,229 pieces) of data are used for repeated verification. The same sorting method is also applied to nocturnal hourly data. Models completed with training would undergo repeated verification with diurnal data.

## 3. Methodology and Network Training

### 3.1. Match-Up Data

To create the match-up data between SSTs derived from satellite infrared sensors and *in situ* measurements, we produced pairs of co-located satellite and *in situ* SST observations with time differences shorter than one hour. The data are converted to be consistent with other data in time scale and then imported into the database. We also compute the daily mean and its standard deviation and then delete the data falling outside three standard deviations to remove the outliers [36]. The same methodology is used in calculating hourly datasets.

A random selection is the most suitable way for an ANN analysis as the training and validation process requires “familiarizing” the network with all possible conditions of inputs and the corresponding target outputs [37–39]. Within the large coverage areas of GOES sensor, more data would be acquired from positions away from the nadir than from those near the nadir. To minimize variables’ weighted difference in model calculation resulted from uneven distribution of data, stratified sampling method on the basis of 0.5 K, a PO.DAAC data bias, are used in the analysis of consolidated hourly mean data (Table 3). The number of randomly sampled SST data does not exceed 1,000 with a bias of 0.5 K in each station during the same month in each year. The number of randomly sampled SST data with a bias greater than 0.5 K or smaller than 0.5 K in each station during the same month in each year does not exceed 1,000, either.

### 3.2. Network Construction

**Figure 2.**
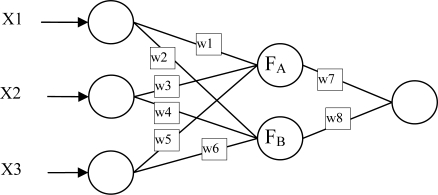
The ANN model.

BPN plays a very important role in the artificial neural network. The reason to improve BPN is to enhance the training speed, avoid falling into local minima, and promote the capability. The traditional BPN adopts the steepest descent method to train and update weight values, and has the following shortcomings: (1) it is apt to converge to local minima; (2) slowly updated weight values and (3) long learning times, possibly causing divergent results. In this study, we combined the advantages of the Steepest Descent Method and the Newton Method in the Levenberg-Marquardt (L-M) as the BPN learning law [38–41]. Three-layer Back Propagation Algorithm (BPA), including input, hidden, and output layer, is employed in this study (Figure 2). There are six hidden neurons. Epochs are 1,000, goal is 0.1, and max fail is 6. The output variable is the TAO SST (*X_sst_*). In the back-propagation neural network, complete connection is established between each input variable and each neuron in the hidden layer, while each connection is given a weight (Figure 2). Through the interconnection between the three weight values, w1 (w2), w3 (w4), w5 (w6) and three input variables, the relative contribution of each input variable to the hidden neuron *F_A_* (*F_B_*) would be determined. The weight of each hidden neuron can be used to determine the most influential variable in the model and can be calculated using Equation (4) as:

(4) $$\left[\begin{array}{ccc} w1 & w3 & w5\\ w2 & w4 & w6 \end{array}\right]=\left[\begin{array}{c} F_{A}\\ F_{B} \end{array}\right]$$

Mean Absolute Percentage Error (MAPE), a relative measure that incorporated the best characteristics among various accuracy criteria [40], would serve as a forecast performance indicator as defined by Equation (5) as:

(5) $$\mathrm{MAPE}=\frac{\sum_{k=1}^{T}\left|\frac{d_{k}-y_{k}}{d_{k}}\right|}{T}\times 100$$

where *d_k_* is the actual value of data number *k*, *y_k_* is the forecast value of data number *k*, and *T* is the total number of data. According to MAPE criterion for the assessment of a model proposed by Lewis [41], a percentage less than 10 represents high forecasting accuracy.

### 3.3. Network Training

**Figure 3.**
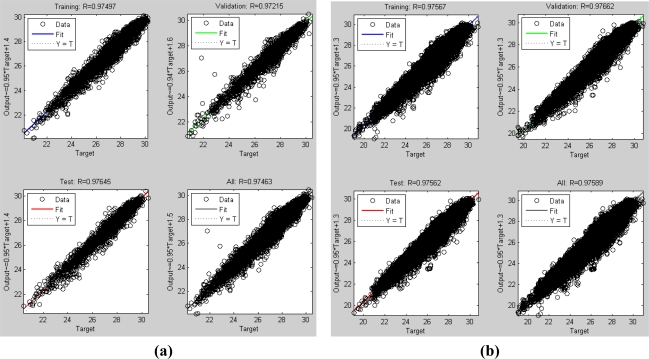

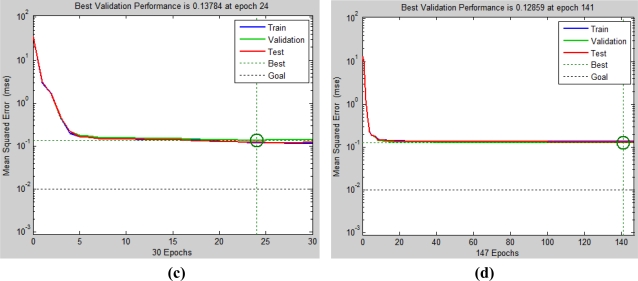
BPN training result by daily mean data **(a, c)**, and by hourly mean data **(b, d)**.

Results of network training are shown in Figure 3. Figure 3(a,c) shows daily mean data with a correlation coefficient (R) of 0.97, Root Mean Square (RMS) of 0.36 K, and MAPE of 1.03%. Figure 3(b,d) shows hourly mean data with R of 0.98, RMS of 0.36 K, and MAPE of 1.3%. The BPN weight tables of daily and hourly mean data are shown in Tables 4 and 5, respectively. Because all the input data for BPN have been normalized in the pre-processing procedure, the coefficients of each hidden neuron (*F_n_*, *n* ∈ 1 ∼ 6) trained with daily and hourly mean data may not be the same. Thus the values of weight table in Tables 4 and 5 are the normalized weights. We can compare the importance with the total weight (or sum) of different input variable. From Tables 4 and 5, atmospheric temperature is the most important factor when deriving SST in the tropical area. The second important factors between the daily and the hourly BPN models are not the same. For hourly mean data, the second factor is $X_{i}^{\mathrm{ws}\_\mathrm{std}}$, which is also the forth factor for daily mean data. The order is raised for hourly data. The reason is that there are daily surface wind variations over the equatorial Pacific Ocean. The semidiurnal zonal wind variations are thought as the atmospheric thermal tide [42–45]. Daily mean data is not sufficient to observe such intraday variations. Except for, other weight orders of variables are almost similar. This indicates that the BPNs have good generation ability and stability of the results for daily and hourly mean data.

**Figure 4.**
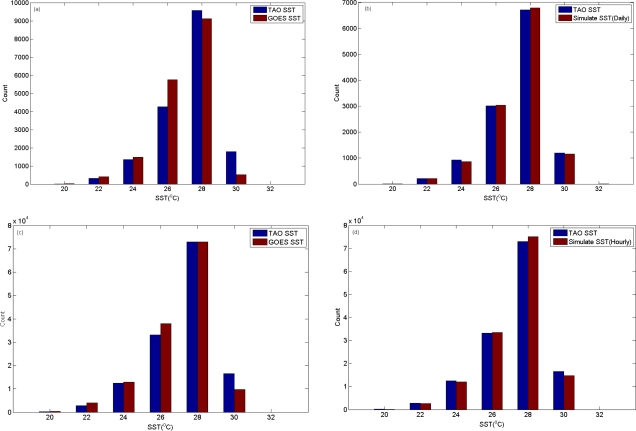
Histogram of original SST and simulated SST. **(a)** Daily data of GOES SST *vs.* TAO SST; **(b)** Daily data of simulated SST *vs.* TAO SST; **(c)** Hourly data of GOES SST *vs.* TAO SST; **(d)** Hourly data of simulated SST *vs.* TAO SST.

Figure 4 shows the daily data and hourly data of GOES SST and the simulated SST *versus* TAO SST. Comparing with TAO SST data, the original GOES SST data exhibited a tendency to overestimate at lower temperatures (<28 °C), and an underestimating tendency at higher temperatures (≥28 °C) (Figure 4(a,c)). However after applying the model, the accuracy of simulated data is significantly improved (Figure 4(b,d)). As shown in the BPN weight table (Tables 4 and 5), $X_{i}^{\mathrm{airt}}$ exhibited higher weight values in both daily and hourly data. A comparison with heavy-weighted variables removed is listed in Table 6 which shows an increase of RMS by more than 0.6 K for both daily and hourly data. When the top three variables of the sum of absolute weight values are removed, the RMS is increased by more than 1 K for daily data and 0.7 K for hourly data. Both $X_{i}^{\mathrm{airt}}$ and $X_{i}^{\mathrm{rh}}$ are the most influential factors in daily and hourly models.

## 4. Results and Discussion

**Figure 5.**
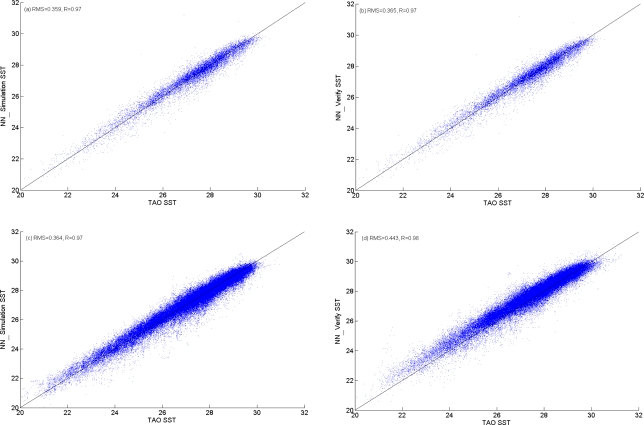
Scatter-plot of daily verification data and hourly verification data with *in situ* measurements.

Sources of errors from satellite-derived SST include aerosol and water vapor in the atmosphere, clouds, instrument errors and the sampling errors from skin effect [13], among which, instrument errors may cause measurement inaccuracy up to 0.1–0.2 K [44]. Many studies with GOES SST employed nocturnal data only, or focused on specific sea areas and time periods [12], with an aim for acquiring potentially better data. This study employs a large amount and continuous in-situ TAO and GOES SST data for analysis, and the data quality of GOES SST is significantly improved. The model established with BPN algorithm reduces RMS from original 0.58 K to 0.38 K for daily data, and from 0.66 K to 0.44 K for hourly data. Repeated the verification of model in Figure 5, the RMS is 0.37 K and the correlation coefficient R is 0.97 for daily data and are 0.44 K and 0.98 for hourly data, respectively. Such results are not only better than the original data error of 0.5 K but also 1 K by Wu *et al.* [36] and 0.7 K by Liu *et al.* [45].

Results of the BPN model successfully improve the accuracy of GOES SST and reveal that air temperature and relative humidity are the two main factors contributing to GOES SST bias. Changes in air temperatures can be translated as the amount of vapor from sea water evaporation, while sea surface wind may also change relative humidity by blowing vapor away because higher wind speeds will assist in evaporation. The movement of vapor may affect the data acquired by satellite infrared sensors and then affect the bias of GOES SST.

Changes in SST are generally determined by net heat flux at sea surface and oceanic mixing processes [46]. SST, unlike land surface temperature, does not exhibit obviously diurnal variations. Its diurnal variation is about 0.5 K, or sometimes up to 2 K [47]. SST rises during daytime in response to solar radiation heating and weaker wind stress, while SST drops at nighttime when net heat flux becomes negative. Such periodic diurnal variations indicate that sea surface is heated up during one-fourth of the day and cools down during the other three-fourths of the day [48]. The BPN model for daily data in this study is constructed using nocturnal data with repeated verification by diurnal data, which increases R from 0.97 to 0.98 and remains RMS at 0.44 K, presenting the ability of bias correction.

$X_{i}^{\mathrm{ws}\_\mathrm{std}}$and $X_{i}^{\mathrm{ag}}$ exhibit more pronounced weight in hourly data analysis than in daily data due to the comparatively smaller time scale. Moreover, infrared signals might be sensibly affected at positions away from the nadir due to longer path.

From Figure 4(a,c), we found that when the SST is 28 °C above, *in situ* SST is higher than the GOES SST, indicating that the GOES SST is underestimated. In analysis of wind speed, we found 62% of the SST data below 28 °C when the wind speed is above 6 ms^−1^. Donlon *et al.* [49] proposed that above a wind speed of approximately 6 ms^−1^ the relationship between the skin SST and bulk SST, is well characterized for both day and nighttime conditions by a cool bias of −0.17 ± 0.07 K rms. Tropical Pacific SST generally maintains in high temperature. When the SST is lower than 28 °C, it mostly contains rainy. The moisture in the atmosphere and clouds absorbing infrared will cause GOES imaging errors. In the hourly data set, the infrared reflects from ocean surface disturbed by more water vapor under the satellite azimuth larger optic depth. Although our results suggest that surface wind speed in not a major contributor to the error budget, the variation of surface wind speed is an important contributor to the error budget (for daily dataset, the ranking of importance is at the fourth order; for hourly dataset, it is at the second order). The BPN method doesn’t work well for the uniform variable. Therefore we can’t retrieve the contribution of the uniform trade wind in the tropical Pacific. However, the method retrieves the variation of surface wind speed as a major contributor. This implies that the wind speed is one of the major sources of errors, which consists with the currently accepted view.

Owing to a lack of ground receiving station of polar-orbiting satellites in the tropical Pacific region and the much longer time interval of viewing the same place than that of GOES, these have given GOES an advantage in long term observing Oceania region. The distance between GOES and the Earth’s surface is longer than that of a polar-orbiting satellite causing inferior ground resolution as well as lower quality of SST data when compared with polar-orbiting satellites. However, our ANN analysis proves the capability of accuracy improvement of GOES SST with its bias correction and points out the factor of latent heat playing a key role on GOES bias.

## 5. Conclusions

This study presents an appropriate BPN algorithm for improving the accuracy of SST product derived from GOES infrared sensors. By using the algorithm, the RMS of GOES daily SST data are reduced from 0.58 K to 0.38 K and the hourly data are from 0.66 K to 0.44 K. The algorithm also reveals that air temperature and relative humidity which may reflect to the latent heat are the major factors to affect the accuracy of SST in the tropical Pacific. From the hourly data analysis, short-term weather changes produced by thin cloud or water vapor may also affect the accuracy of SST products.

BPN architecture is not only simple but also has good data predictability. Many previous studies have used this algorithm. However, the main disadvantages of BPN are its easily involvement in local convergence and slowness. In order to make the analysis efficient, we use the L-M method to accelerate the learning speed of the BPN. Besides, a second sample of data validation eliminates the local convergence problem. The result of model analysis is stable and closes to the result of verification. Limited *in situ* data, SST and wind speed relationship require further analysis under 28 °C. Future studies are expected to apply the model to estimate other infrared sensors’ products.

## Figures and Tables

**Table 1.** List of predictor variables.

	**Daily input data**		**Hourly input data**
$X_{i}^{\mathrm{airt}}$	Air Temperature	$X_{i}^{\mathrm{airt}}$	Air Temperature
$X_{i}^{\mathrm{ag}}$	The satellite’s azimuth angle	$X_{i}^{\mathrm{ag}}$	The satellite’s azimuth angle
$X_{i}^{\mathrm{rh}}$	Relative humidity	$X_{i}^{\mathrm{rh}}$	Relative humidity
$X_{i}^{\mathrm{rh}\_\mathrm{std}}$	Standard deviation of relative humidity	$X_{i}^{\mathrm{rh}\_\mathrm{std}}$	Standard deviation of relative humidity
$X_{i}^{\mathrm{wspd}}$	Wind speed	$X_{i}^{\mathrm{wspd}}$	Wind speed
$X_{i}^{\mathrm{ws}\_\mathrm{std}}$	Standard deviation of wind speed	$X_{i}^{\mathrm{ws}\_\mathrm{std}}$	Standard deviation of wind speed
$X_{i}^{\mathrm{wd}}$	Wind direction	$X_{i}^{\mathrm{wd}}$	Wind direction
$X_{i}^{\mathrm{nlwrs}}$	Net longwave radiation flux		

**Table 2.** Number of data used for training and verification.

	
	**Daily data**	**Hourly data**
Training	12,074	138,028
Verification	5,229	108,426
Total	17,303	246,454

**Table 3.** The number of original data and clean data.

	
	**Original data**	**Clean data**
**Daily dataset**	35,102	17,303
**Hourly dataset**	1,326,043	246,454

**Table 4.** BPN weight table of daily mean data.

	$X_{i}^{\mathrm{ag}}$	$X_{i}^{\mathrm{airt}}$	$X_{i}^{\mathrm{wspd}}$	$X_{i}^{\mathrm{ws}\_\mathrm{std}}$	$X_{i}^{\mathrm{rh}\_\mathrm{std}}$	$X_{i}^{\mathrm{rh}}$	$X_{i}^{\mathrm{wd}}$	$X_{i}^{\mathrm{nlwrs}}$
F_1_	0.16^*^	0.20^*^	0.02	0.09	0.10	0.08	0.10	0.27^*^
F_2_	0.01	0.47^*^	0.01	0.14^*^	0.24^*^	0.03	0.07	0.03
F_3_	0.01	0.10^*^	0.01	0.03	0.57^*^	026^*^	0.01	0.02
F_4_	0.04	0.38^*^	0.01	0.02	0.18^*^	0.33^*^	0.04	0.02
F_5_	0.03	0.50^*^	0.03	0.03	0.19^*^	0.22^*^	0.00	0.00
F_6_	0.15^*^	0.05	0.03	0.18^*^	0.07	0.35^*^	0.10	0.06

Sum	0.39	1.70	0.11	0.49	1.35	1.26	0.31	0.39

Order	6	1	8	4	2	3	7	5

* The top three variable weights in the network.

**Table 5.** BPN weight table of hourly mean data.

	$X_{i}^{\mathrm{ag}}$	$X_{i}^{\mathrm{airt}}$	$X_{i}^{\mathrm{wspd}}$	$X_{i}^{\mathrm{ws}\_\mathrm{std}}$	$X_{i}^{\mathrm{rh}\_\mathrm{std}}$	$X_{i}^{\mathrm{rh}}$	$X_{i}^{\mathrm{wd}}$
F_1_	0.02	0.01	0.00	0.13*	0.60*	0.16*	0.07
F_2_	0.08	0.32*	0.04	0.17*	0.09	0.26*	0.04
F_3_	0.13*	0.31*	0.09	0.34*	0.02	0.03	0.08
F_4_	0.05	0.56*	0.00	0.21*	0.02	0.15*	0.01
F_5_	0.04	0.27*	0.02	0.22*	0.24*	0.19	0.02
F_6_	0.02	0.44*	0.06	0.02	0.11	0.13*	0.21*

Sum	0.34	1.91	0.22	1.10	1.08	0.92	0.43

Order	6	1	7	2	3	4	5

* The top three variable weights in the network.

**Table 6.** Statistical results of model variables.

	**Daily_RMS**	**Hourly_RMS**	**Daily_R**	**Hourly_R**
Original model	0.36	0.36	0.97	0.97
Without $X_{i}^{\mathrm{airt}}$	1	0.97	0.78	0.81
Without $X_{i}^{\mathrm{rh}}$	0.38	0.39	0.97	0.96
Without $X_{i}^{\mathrm{airt}}$ & $X_{i}^{\mathrm{rh}}$	1.08	1.04	0.73	0.79
Without $X_{i}^{\mathrm{rh}\_\mathrm{std}}$	0.38	0.38	0.97	0.96
Without $X_{i}^{\mathrm{wspd}}$	0.35	0.41	0.98	0.96
Without $X_{i}^{\mathrm{airt}},\;X_{i}^{\mathrm{rh}},\;X_{i}^{\mathrm{wspd}}$		1.13		0.71
Without $X_{i}^{\mathrm{airt}},\;X_{i}^{\mathrm{rh}\_\mathrm{std}},\;X_{i}^{\mathrm{rh}}$	1.31		0.53	
